# Enhancing the classification of seismic events with supervised machine learning and feature importance

**DOI:** 10.1038/s41598-024-81113-7

**Published:** 2024-12-24

**Authors:** Eman L. Habbak, Mohamed S. Abdalzaher, Adel S. Othman, HA Mansour

**Affiliations:** 1 National Research Institute of Astronomy and Geophysics, ENDC Department, Cairo, 11421 Egypt; 2 National Research Institute of Astronomy and Geophysics, Seismology Department, Cairo, 11421 Egypt; 3 Electrical Engineering Department, Shobra Faculty of Engineering, Benha University, Cairo, 11629 Egypt

**Keywords:** Machine learning, Seismic discrimination, Earthquakes, Quarry blasts, Feature Importance, Natural hazards, Engineering, Solid Earth sciences

## Abstract

The accurate classification of seismic events into natural earthquakes (EQ) and quarry blasts (QB) is crucial for geological understanding, seismic hazard mitigation, and public safety. This paper proposes a machine-learning approach to discriminate seismic events, particularly differentiating between natural EQs and man-made QBs. The core of this study is to integrate different features into a unified dataset to train some linear and nonlinear supervised machine learning (ML) models. The proposed approach considers a collection of 837 events (EQs and QBs) with local magnitudes of $1.5 \le M_{L} \le 3.3$ from the Egyptian National Seismic Network (ENSN) seismic event catalog between 2009 and 2015. This paper’s principal contribution is applying feature selection techniques and feature importance analysis to identify the best features leading to the best events’ discrimination. In other words, the proposed approach enhances classification accuracy and provides insights into which features are most crucial for distinguishing between EQ and QB events. The results show that with only three features, corner frequency, power of event, and spectral ratio, the best-developed ML model accomplishes a discrimination accuracy of 100% among several benchmarks of linear and non-linear models.

## Introduction

The northern region of Egypt is known for its many artificial seismic sources, mostly due to mining activities and the cement industry. While Central Egypt also experiences some artificial seismic activities, they are not as frequent as in the north. These activities create artificial seismic vibrations, which obviously are recorded by the Egyptian National Seismic Network ENSN. The primary role of the ENSN is to monitor seismic events, and its stations cover the entire country. However, these artificial blasts can cause contamination of the EQ catalog, leading to an inaccurate evaluation of seismic hazards and risk studies. Therefore, it’s important to discriminate between small EQs and artificial seismic events like quarry blasts QBs^1–3^. Discriminating between natural and artificial seismic events is a significant challenge in seismology. Historically, seismologists spent time and effort to discriminate between these two types of events. Taking this into account, identifying, discriminating, and eliminating artificial seismic activities is an essential initial phase in the analysis that relies on the catalogs of seismicity^4–6^. This process is crucial for a better understanding of EQs and the development of more effective seismic monitoring systems. Notably, these artificial seismic disturbances are typically of low magnitude, which makes the discrimination process more complex. Various techniques and parameters have been developed to discriminate EQs and artificial activities, focusing on extracting signal characteristics in both the time and frequency domains. Among these techniques are amplitude peak ratios^7–9^, spectral ratios of seismic phases^10,11^, P/S corner frequencies^12^, and various other methods have been employed for this issue^13–15^. Other algorithms based on mathematical criteria were used to clear up regional catalogs and remove artificial activities^16–18^. In recent years, machine learning (ML) methods have become more influential in the area of seismology where they have enabled the processing of more data with less effort. These methods are adept at identifying and differentiating events based on their unique signatures, making them valuable for a range of monitoring objectives^18–20^. Linear models like logistic regression (LR), linear discriminant analysis (LDA), and affinity propagation^21–23^have been applied in classification tasks, though they have certain limitations in efficiency. On the other hand, models like Naive Bayes (NB)^22^, random forest (RF)^24^, and support vector machine (SVM)^25,26^have shown enhanced efficiency. Moreover, some other studies have used multiple modes for discrimination purposes^18,27–29^. From Egypt, recent research has focused on the discrimination of EQs and QBs with ML techniques. A study that used SVM with wavelet showed a valuable accuracy^30^, while others have explored the use of advanced methods like scalogram, capsule, and convolutional neural networks for this purpose^31,32^. Globally, deep learning approaches have also taken place in seismic source discrimination research^33–35^. In addition, these algorithms have been used for the discrimination of nuclear explosions^36^. Some other studies concentrated on using the ResNet network to classify seismic and blast events^37,38^. As ML and its tools continue to develop, there is a growing focus on developing high-performance models that require minimal data. Thus, this work concentrates on using feature importance and feature selection methods to reduce the amount of data needed to build an effective ML classifier. The approach aims to develop an ML model for classifying EQs and QBs using a limited amount of training data. This research specifically utilizes data from Northeastern and Central Egypt, analyzing features like complexity (C), event power (P), the S-wave to P-wave peak amplitude ratio (As/Ap), corner frequency (Fc), seismic moment(Mo), moment magnitude (Mw), and the spectral amplitude ratio (Sr)

**Fig. 1:**
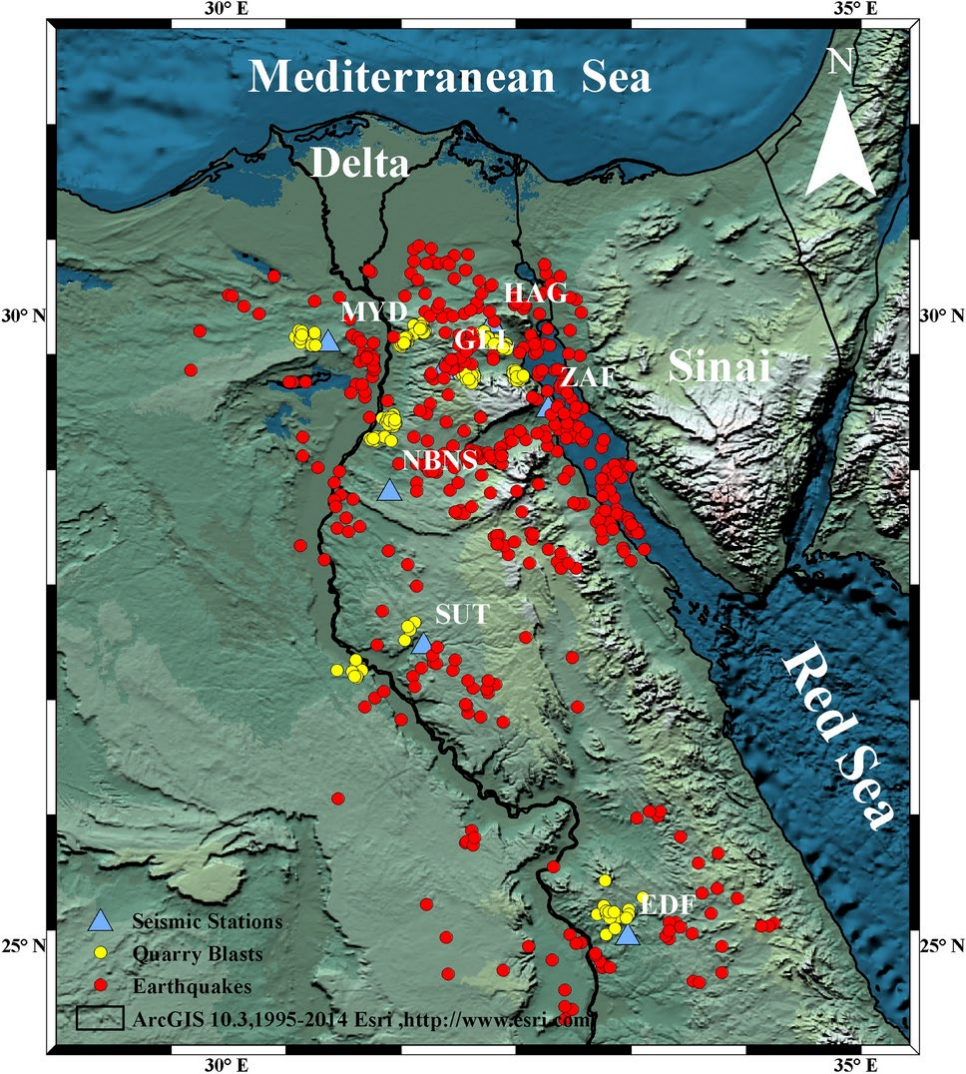
Geographical distribution of selected events and the ENSN seismic stations (Done by ArcGIS 10.3,1995–2014 Esri, http://www.esri.com).

from seismographic records. Our study presents a novel approach by utilizing feature importance and feature selection methods. Unlike previous research studies in this field, our work specifically implements these techniques to the extracted features of seismic events. This process is significant as it enhances the accuracy and efficiency of classification models by identifying and choosing the most relevant features in seismic data. Therefore, our research determines the best parameters that can be used in the discrimination of EQs and QBs. Key contributions of this research include: (1) Introducing a classification method that reduces feature count using feature importance and feature selection techniques, effectively separating EQs from QBs. (2)The model operates with just three features derived from data collected across seven stations in Northern and Central Egypt. (3) The approach employs various linear and nonlinear ML techniques, identifying the most effective one for differentiating EQs and QBs, thus potentially improving seismic hazard predictions and enabling quicker EQ identification to mitigate their impact. (4) The method’s effectiveness is evaluated using various metrics, including accuracy, Cohen Kappa score, F1-score, Matth correlation coefficient, and others. In the next section, the used methodology is presented in detail, focusing on all the main aspects, from data collection and preprocessing to the proposed workflow, training, and testing. Then, in the “Discussion and results” section, these are shown and discussed. The final section is left to the conclusions. Here, we define the abbreviations used throughout the paper Table 1.

## Methodology

### Experiment setup

- **Data Collection**

**Fig. 2:**
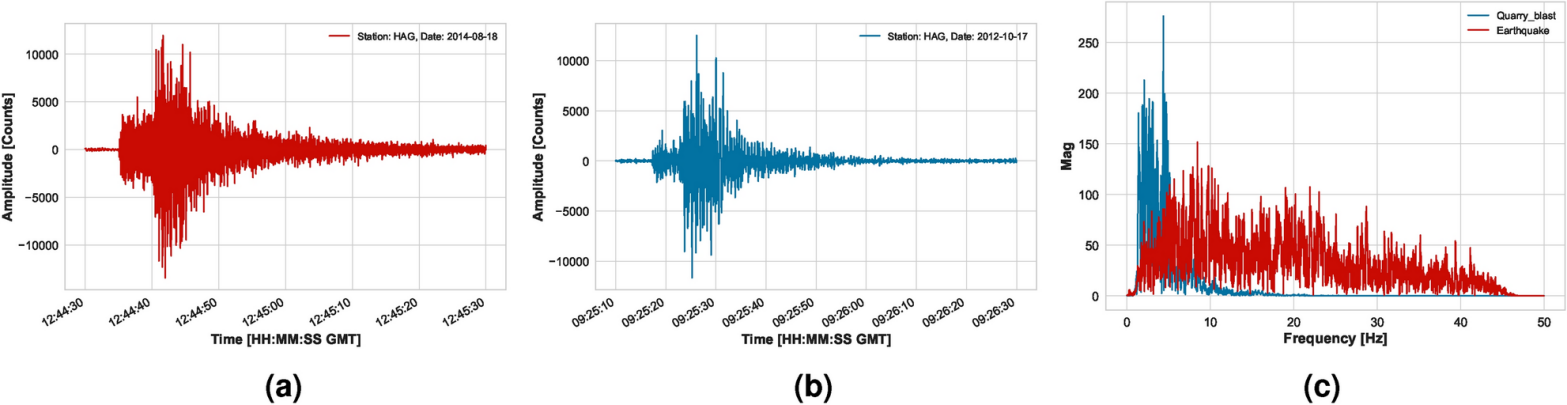
Representation of a typical EQ and QB in time and frequency domains.

In the study, we employed a classification process to differentiate between earthquakes (EQ)s and (QB)s recorded by seven distinct seismic stations across Northern and Central Egypt. The data was thoroughly gathered from the HAG, ZAF, MYD, GLL, EDF, and SUT short-period stations, and the NBNS broadband station, chosen for their clear event recordings and absence of technical issues. Our research is built on a dataset comprising 837 seismic events from 2009 to 2015, which include local magnitudes$1.5 \le M_{L} \le 3.3$. This dataset incorporates 434 EQs and 403 QBs, all occurring in Northern and Central Egypt. The geographic spread of these events and the involved stations are illustrated in Figure 1 created using ArcGIS software. Data from both short-period and broadband stations of the ENSN were precisely extracted from the database, focusing on EQs with depths less than 25 km. The identification of most QBs was achieved through monthly quarry reports received by the ENSN’s main center, independent of seismic methods. Additionally, Figure 2presents a time and frequency representation of a typical EQ and QB signal. This work is based on and extends upon waveform analysis and signal characteristic studies from earlier research^39,40^. Only the accurately localized seismic events data from the ENSN Catalog were reviewed and manually analyzed to identify the clear P and S. This was done on waveforms with a good signal-to-noise ratio and well-defined arrivals for both P and S waves. The window length of the p-wave ranges from 1.5 to 4 seconds, while it ranges from 10 to 20 seconds for S-wave, depending on the distance.The automatic classification of these events was based on various collected features extracted from the chosen seismograms. From the gathered data, 51.85% are classified as EQs, while the remaining 48.148% are QBs, totaling 837 events. As a result, several key values have been computed for the different input features, as detailed in Table 2. This table provides a comprehensive overview of the descriptive statistics of the collected data, including central tendency measurements such as the mean and median and variability measures like the first and third quartiles (Q1 and Q3). These quartiles are statistical parameters that describe how the data is split into four quarters to give an idea about the distribution of the data values. The features in this table can be described as follows:

**Corner frequency (Fc)**:The corner frequency is a critical threshold in the frequency response at which energy flowing through the signal starts to diminish rather than pass freely. It can also be described in the Fourier spectrum as the intersection point between the low- and high-frequency asymptotes as widely defined in seismological studies^41–43^. It was estimated using the EQK_SRC_PARA software^44^.

**The seismic moment (Mo)**: It is a physical quantity representative for energy released during the seismic event. It mainly based on the estimation of low-frequency spectrum plateau and can be defined by^45^::

1 $$\begin{aligned} Mo =\frac{4 \pi \rho V^3 \Omega _0 R}{F *\Re \theta \varphi }, \end{aligned}$$

where $\rho$ is rock density,*V* represents velocity, $\Omega _0$ is the low-frequency spectral level, *R* is the epicentral distance, *F* is the free surface effect, and $\Re \theta \varphi$ represents the radiation pattern. The free surface effect *F*is assumed to be 2, as noted by Snoke^46^, while the average radiation pattern coefficients are 0.64 for P-waves^43^and 0.55 for S-waves^47^. The low-frequency spectral level $\Omega _0$is estimated using the EQK_SRC_PARA software^44^.

**The moment magnitude (Mw)**: It is estimated according to Hanks and Kanamori concept^45^ as

2 $$\begin{aligned} Mw = \frac{2}{3} \log (Mo) - 10.73 , \end{aligned}$$

where *Mo* is the seismic moment.

**Event complexity (C)**: It is the ratio of the power of velocity vertical component of the event *S*(*t*)in two different time frames represented by^48^:

3 $$\begin{aligned} C = \frac{\int _{t_1}^{t_2} S^2(t) dt }{ \int _{t_0}^{t_1} S^2(t) dt}, \end{aligned}$$

where $t_0$ is the onset time of P-wave.

**Peak Amplitude ratio(As/Ap)**: It is the ratio of the maximum amplitude of S -wave (As) to the maximum amplitude of P- wave (Ap).

**Spectral ratio (Sr)**: it is the ratio of the event’s amplitude spectra in the high-frequency band ( $h_2-h_1$) to the low-frequency band ($l_2-l_1$) specified as^49^:

4 $$\begin{aligned} Sr = \frac{\int _{h_1}^{h_2} a(f) df}{ \int _{l_1}^{l_2} a(f) df}, \end{aligned}$$

where *a*(*f*) is event’s spectral amplitude. The high-frequency band ( $h_2-h_1$) were identified as (10-5 )Hz and low-frequency band ($l_2-l_1$) as (5-1) Hz, based on the analysis of spectra from all seismograms in Northern Egypt. In Central Egypt, the chosen high-frequency band ( $h_2-h_1$) was (14-7) Hz, while the low-frequency band ($l_2-l_1$) was (7-1) Hz.

**The event’s power (Pe)**: It is described by^14^:

5 $$\begin{aligned} Pe = \left( \frac{As}{Ap}\right) ^2 \times C \times (Sr)^2, \end{aligned}$$

and it was used in our study in a logarithmic case as some studies used it^14,40,50^.

**Table 1:** Table of Abbreviations.

Abbreviation	Definition
AB	AdaBoost
CB	CatBoost
DT	Decision Trees
ENSN	Egyptian National Seismic Network
EQ	Earthquake
ET	Extra Trees
GB	Gradient Boosting
GNB	Gaussian Naive Bayes
KNN	k-Nearest Neighbor
LDA	Linear Discriminant Analysis
LR	Logistic Regression
LSVM	Linear Support Vector Machine
ML	Machine Learning
NB	Naive Bayes
QB	Quarry Blast
RF	Random Forest
SMOTE	Synthetic Minority Over-sampling Technique
SVM	Support Vector Machine

- **Preprocessing of relevant dataset**

**Fig. 3:**
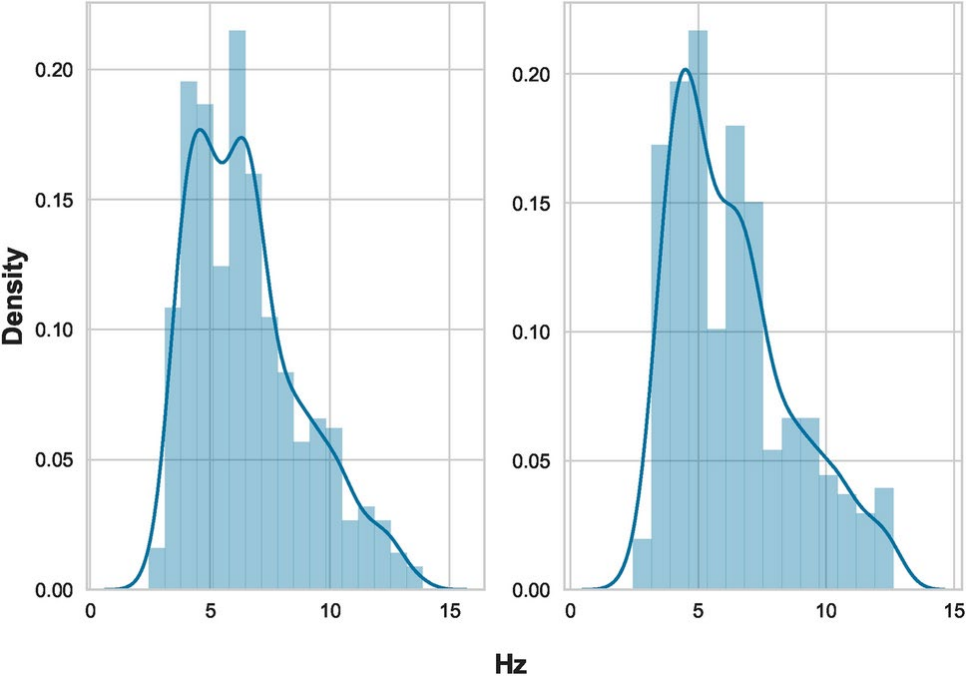
Distribution of a feature Fc(p) pre- and post-outlier elimination.

**Fig. 4:**
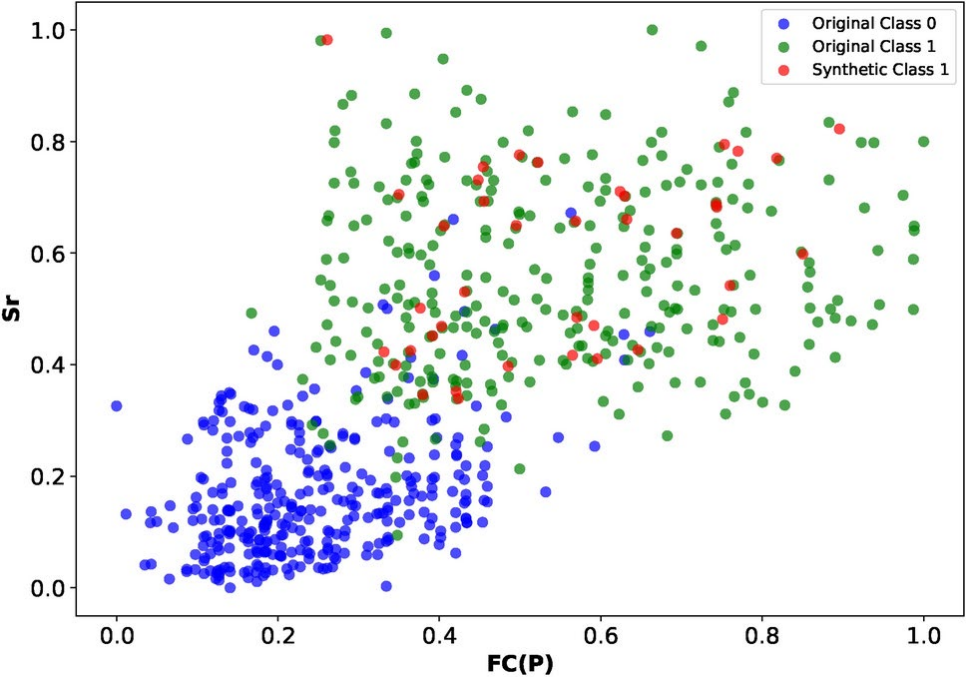
A view of generated synthesized samples of minority class with respect to original classes’ points of features Sr and Fc(p).

In our study, we address a binary classification issue that involves differentiating between natural and artificial seismic events recorded by the ENSN. The datasets include features indicative of potential seismic event types, categorizing them as either tectonic (EQ) or non-tectonic (QB). These features, derived from various previous studies, were organized according to different recording stations. We combined all files into a single dataset, aligning them by event location and time, and introduced a new label column: ’1’ for EQ and ’0’ for QB. Consequently, each event is represented by a row containing various extracted features and its class label (0 or 1), which include corner frequency, seismic wave amplitudes, complexity, spectral ratio, moment magnitude, seismic moment, and event power. Accordingly, these features are implemented as the training input matrix X, while the target vector y is represented by the class label variable. It is common for real-world seismic activity data to be inherently disordered. An essential first step before analyzing such data is its refinement, which involves correcting the data structure and eliminating noise. In our research, we have implemented a series of preprocessing steps. Initially, the dataset had some missing (NAN) values observed, so iterative imputation^51^was implemented.As mentioned before, there were several data tables that were collected, merged, and combined. The missing values were few, and the imputation did not affect the data distribution. Further data examination led to the effective removal of outliers. Outliers represent the data points or values that differ significantly from the majority of points in a dataset’s distribution. These points usually have high or low values compared to other observations and may arise for various reasons. In ML, identification and dealing with outliers is an important step in data preprocessing and cleaning. Outlier removal can ensure that the data is more representative, which improves the reliability and validity of the analysis. Many methods are used for outlier estimation, like IsolationForest, Local Outlier Factor (LOF), and Interquartile Range Method (IQR)^52–54^. We used (IQR) since it is simple and easy to apply. It calculates the range between the 25th and 75th percentiles (Q1) and (Q3), respectively, and identifies a threshold range of $(Q1 - 1.5 \times IQR , Q3 + 1.5 \times IQR)$. Outliers are those data points that fall outside that range. This method was applied using a Python code to each column in the features data set, and the distribution was plotted before and after the removal. Figure 3shows a comparative view of the distribution of one feature (Fc(p)). In the right subplot, which shows the data after outlier removal, the data distribution is more centralized and less skewed than the left one. This means that the outliers were effectively identified and removed, which leads to a more representative feature distribution. By removing these outliers, the model can focus on learning patterns from the core distribution of the data and achieve better performance. After that, the dataset undergoes normalization to ensure all dimensions are on a comparable scale. This step enhances the accuracy of data processing by taking into account the variance between different dimensions. Post-outlier removal, we observed data imbalance, which was rectified using the SMOTE method from the Imblearn library. This method is effective because it generates plausible new synthetic examples from the minority class that are relatively similar to existing examples in the feature space. SMOTE is considered to be the most widely used method for generating synthetic examples. It was introduced by Nitesh Chawla, et al^55,56^. to address the class imbalance by creating additional examples for the minority class. It works by finding the nearest neighbors in the feature space and drawing a line between these features, creating a new point on that line. This ensures that the new examples are consistent with the existing data pattern and don’t deviate away from the majority or affect the data distribution. With review and examination of these examples, they were found reasonable. This was confirmed by visualizing the data distribution after applying the method. As can be seen in figure Figure 4of two features Sr, Fc(p) data points, the synthesized examples did not change the overall data distribution. They appear to be placed within the feature space between existing data points in a way that they mimic these points of the minority class. Additionally, during feature engineering^57^, we eliminated highly correlated features to enhance the discriminative power of the dataset. This was performed using Python libraries Numpy and Pandas to measure the correlation between columns in the data sets.

**Table 2:** Descriptive statistics of features used in ML experiment.

Statistic	Type	Count	Mean	Std	Min	Q1	Median	Q3	max
	EQ	434	8.17	2.02	4.78	6.47	7.64	9.50	13.88
	QB	403	4.81	1.06	2.44	3.99	4.66	5.43	8.50
**Fc(S)**	EQ	434	6.37	1.67	4	5.08	6.11	7.45	11.32
	QB	403	2.48	0.70	1.37	1.87	2.41	3.05	4.33
**Mw(S)**	EQ	434	2.22	0.34	1.52	1.99	2.22	2.43	3.20
	QB	403	2.22	0.34	1.52	1.93	2.21	2.46	3.11
**Mw(P)**	EQ	434	2.28	0.33	1.51	2.05	2.27	2.48	3.31
	QB	403	2.20	0.34	1.51	1.92	2.20	2.45	3.11
**Mo(P)**	EQ	434	6.20e+12	1.10e+13	2.40e+11	1.40e+12	3.10e+12	5.90e+12	1.20e+14
	QB	403	4.40e+12	6.30e+12	1.30e+11	8.70e+11	2.10e+12	5.10e+12	4.70e+13
**Mo(S)**	EQ	434	5.20e+12	9.20e+12	2.20e+11	1.00e+12	2.20e+12	4.90e+12	7.40e+13
	QB	403	4.50e+12	6.40e+12	1.60e+11	8.80e+11	2.20e+12	5.40e+12	5.80e+13
**C**	EQ	434	3.23	1.36	0.53	2.61	2.93	3.44	12.05
	QB	403	1.59	0.70	0.17	1.07	1.50	1.99	4.78
**Sr**	EQ	434	1.92	0.62	0.91	1.45	1.56	2.28	4.03
	QB	403	0.79	0.44	0.10	0.45	0.69	1.21	3.22
**Ap**	EQ	434	5044.22	15374.61	139	1904.50	3979.45	4493.81	287413
	QB	403	3379.02	5175.71	187	882	2297	4042.19	71099
**As**	EQ	434	8731.17	21220.92	466	4200	5683.52	7401.90	350000
	QB	403	2927.93	3164.27	172	797.50	1886	5197	36870
**As/Ap**	EQ	434	2.05	0.79	0.68	1.56	1.59	2.46	5.77
	QB	403	0.98	0.37	0.11	0.71	0.93	1.30	2.10
**(As/Ap)**⌃**2**	EQ	434	5.16	4.05	0.47	3.24	3.28	6.07	33.32
	QB	403	1.26	1.05	0.01	0.51	0.86	1.68	4.40
**Sr**⌃**2**	EQ	434	4.39	2.63	0.83	2.87	2.93	5.19	16.27
	QB	403	0.95	1.09	0.01	0.21	0.48	1.47	10.39
**log(Pe)**	EQ	434	1.35	0.74	−0.04	0.65	1.20	2.04	3.34
	QB	403	−0.34	0.67	−2.42	−0.86	−0.32	0.25	1.23

### Proposed workflow and employed ML classifiers

In this research, we aim to automate the process of data classification, which traditionally requires human intervention to determine the source type. Specifically, we propose a classification approach that discriminates EQs and QBs using ML models. This approach is designed to use feature importance and feature selection to minimize the amount of training data, yet it is efficient at recognizing highly variable event patterns. We begin with a brief comparison of linear and nonlinear types and then detail our proposed approach, which is based on these models. Here, we define the frequently used notations throughout the paper Table 3

- **Employed Linear Models**

1. Quadratic and linear discriminant analysis: LDA proves more effective than LR with datasets where the size of a sample is small, and the predictors are normally distributed. If the data deviates from linear assumptions, LDA becomes insufficient. In such scenarios, Quadratic Discriminant Analysis (QDA) becomes more suitable than LDA^58^.
2. Logistic regression: LR uses a logit function to model the posterior probability of K feature classes, evaluate, and yield a binary output $\in${0, 1}. This process is essential for determining the likelihood of each feature class grouping^59^.
3. Ridge classifier: The Ridge Classifier is typically used to map label data within the range of $[-1,1]$. It solves classification problems with a regression-based logic^60^.
4. Naive bayes: A special variant of NB designed for handling features with continuous values is Gaussian NB (GNB). In this case, these features are assumed to follow a Gaussian distribution^61,62^. It infers class membership relying on the class’s probability as illustrated:
  6 $$\begin{aligned} p(w=G|CL)=\frac{1}{\sqrt{2\pi \sigma ^2}}\exp {-\frac{(G-\mu )^2}{2\sigma ^2}}, \end{aligned}$$
  where *G* is the probability density, *CL* is the class, *w* is a consistent data input factor, $\sigma$ is the variance, *CL* is the class, $\mu$ is the mean.
5. Linear support vector machine:. The enhanced Linear SVM (LSVM) begins with the result of a first-degree equation, similar to LR, then yields values within the range of $[-1,1]$. Subsequently, the margin between data points and the hyperplane is increased utilizing a cost function equipped with a parameter of regularization. Afterward, the cost function is decreased while weights are updated for gradient calculation. In case of misclassification, the gradient is adjusted by changing the regularization parameter’s loss^63,64^.
6. CatBoost:Yandex developed CatBoost (CB) as an ML technique that is founded on decision trees (DT) and gradient boosting (GB) frameworks.

**Table 3:** Table of Notations.

Notation	Definition
Fc	Corner frequency
Mo	Seismic moment
Mw	Moment magnitude
C	Complexity
Sr	Spectral ratio
As/Ap	Peak amplitude ratio
Pe	Power of event
$\rho$	rock density
*V*	Velocity
$\Omega _0$	Low-frequency spectral level
*R*	epicentral distance
*F*	Free surface effect
*G*	The probability density
*CL*	The class
*w*	Constant data input factor
$\sigma$	The variance
$\mu$	The mean
$T_{n}$	The trees’ number
$U_{k}$	The *k*th regression tree
$S_{n}$	The samples’ number
$\hat{y}_{k}$	The predicted target
$y_{k}$	The classifier output
*I*	The indicator function
TP	True positives
FP	False positives
TN	True negatives
FN	False negatives
$T_{PR}$	The true positive rate
$F_{PR}$	The false positive rate

- **Non-Linear Classification Models**

1. Light gradient, gradient, and extreme gradient boosting classifiers: GB identifies and addresses the weaknesses of classifiers using loss function and high-weight data points. This enables GB to customize generic and specific cost functions for optimized solutions tailored to particular classification issues^65^. Following this, Light GB (LGB) is an evolution of GB as it is more efficient^66^. Lastly, Extreme GB (XGB) is an update of the GB library that is more efficient, versatile, and portable. XGB excels in quick and effective parallel tree boosting^67^.
2. Random forest, extra trees, and decision tree: RF incorporates multiple parallel learners working in tandem to reduce both bias and variance in predictions^68^, it relies on this equation:
  7 $$\begin{aligned} \hat{Y}(r)=\frac{1}{T_{n}}\sum ^{T_{n}}_{k=1}{U_{k} (r)}\ , \end{aligned}$$
  Where $T_{n}$ is the trees’ number, $U_{k}$ is the *k*th regression tree, and *r*isthe input vector. The extra-trees (ET) model is more advanced than the RF model. Moreover, it can deal with difficult problems by breaking them down into several simpler ones with the goal of easier solution achievement^69,70^.
3. K-nearest neighbor: Acting as a decision-boundary-oriented classifier, the k-nearest neighbor (KNN) algorithm assigns an input class to the predominant class among its k-closest neighbors in the feature space^71^.
4. Adaboost: AB is an algorithm that uses adaptive boosting and weighted majority votes to enhance the performance of weak classifiers^72^. As a result, the ultimate model is a composite of these classifiers that can be refined through a series of iterations for a specified number of times^65^.

**Fig. 5:**
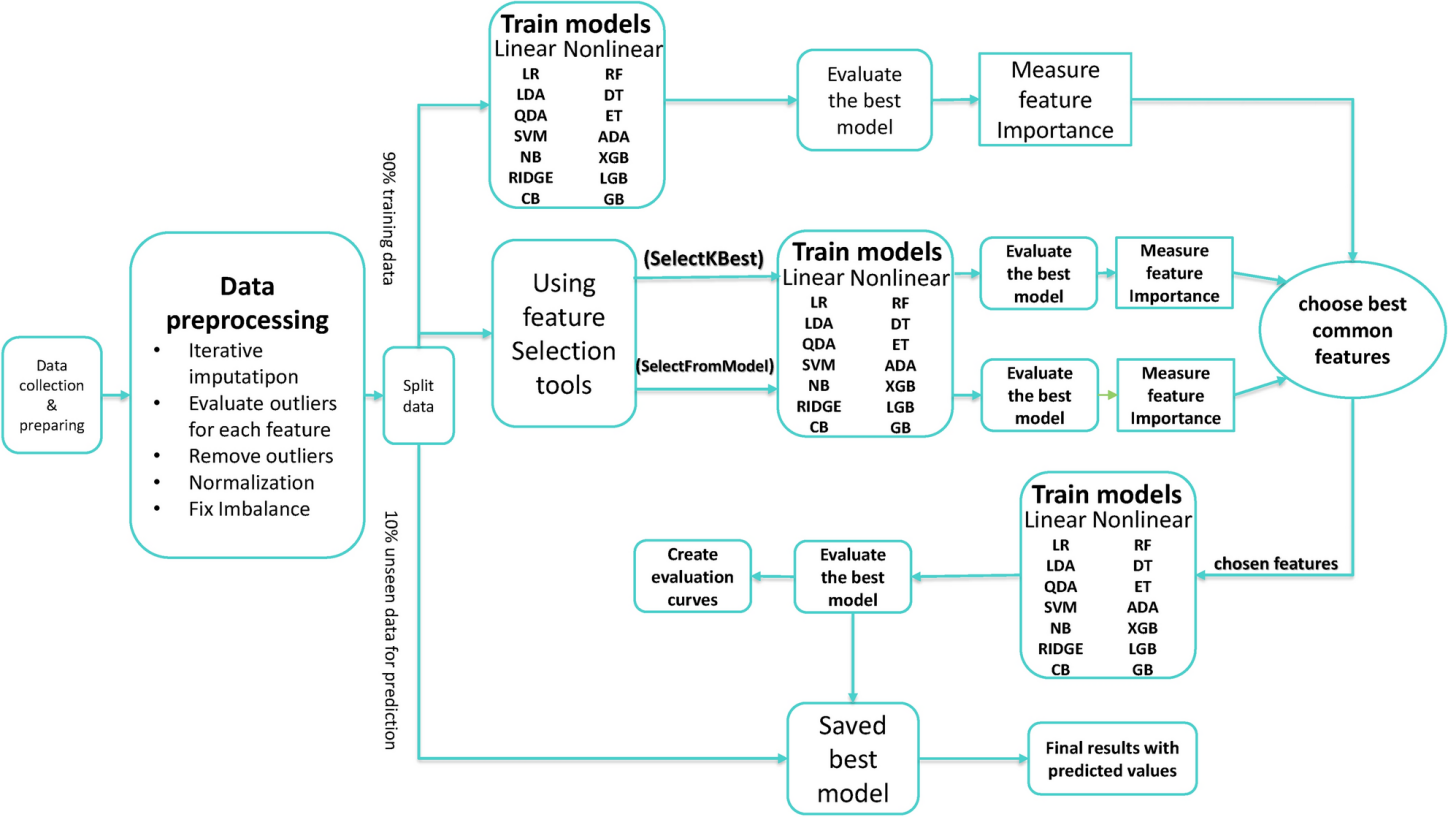
Flowchart of the proposed workflow.

- **Proposed workflow**

Our proposed method integrates both linear and nonlinear ML techniques, as depicted in Fig. 5. This approach involved a comprehensive evaluation using the models shown in the same Figure. Initially, the process began with arranging, merging, and labeling the data of the extracted features. These features include 13 different ones. After preprocessing, the data is split into 90% to work on and 10% for the final prediction. Initially, all features were employed to train and evaluate both linear and nonlinear ML models. For an effective assessment of these models, we employed three different training-to-testing dataset ratios (70%−30% and 80%−20%). The feature importance was obtained for the best-performing model in this first phase. Feature importance means the method that gives scores to the features for their contribution to the ML model’s prediction. These scores are relative, meaning it is like ranking the features according to their effect on the model performance. There are various techniques to measure feature importance, including the Permutation-based method, the coefficient method, and the Decision Trees-based method^73,74^. While the permutation method is available in the Scikit-Learn, it has a large computational time. In our work, we relied on “coef_” and “feature_importance_” attributes after fitting the model. They give feature scores that represent how they affect the model’s prediction. The coef_ attribute is used with linear models, while the “feature_importance_” attribute is used with tree and ensemble models. Then, visualizing these scores through plots and comparing them gave us an idea of the most important features. In parallel, two feature selection methods, ‘SelectFromModel’ and ‘SelectKBest’, are used for selecting five features. Feature selection involves choosing the most relevant independent variables that have the greatest impact on predicting the target variable. The ‘SelectFromModel’ method is a feature selection technique that works by selecting a subset of features based on the importance assigned to them by an underlying model. It is a useful tool that is available in the Scikit-learn library. It works with an estimator to choose features with an importance score above specific threshold criteria or with a defined maximum number of features^75^. We choose the underlying model to be the extreme gradient boosting (XGB) and max_features to five. On the other hand, ‘SelectKBest’ is a feature selection method that selects the top k features with the highest scores based on univariate statistical tests. It chooses the highest k-features scores according to these tests. It is also a Scikit-learn library tool in which we selected the statistical chi-squared test and the number of features to five. Again, we applied both linear and nonlinear ML models across the same dataset split ratios, and the feature importance scores were plotted. At this stage, the most effective features from all methods were selected using the available feature importance plots, the top three common in all plots, which are Sr, Fc(p), and log(Pe). These three features are used to train both linear and nonlinear ML models across the same dataset split ratios, and the best-performing model is obtained. The evaluation metrics of the best-performing model were evaluated while applying kfold cross-validation with seven folds. In the last stage, the 10% unseen part of the data is used with the best features, and the best-performing model and evaluation metrics are evaluated.

### Performance evaluation metrics

Advancements in data storage and computational power have significantly enabled more extensive analysis than was previously possible. Therefore, we employ various metrics to evaluate the effectiveness of our classifiers listed below:

- **Accuracy**

The performance of a model is primarily measured by its accuracy score represented by:

8 $$\begin{aligned} Accuracy = \frac{\sum ^{S_{n}}_{k=1} I(\hat{y}_{k}=y_{k})}{S_{n}}\ , \end{aligned}$$

where $S_{n}$ is the samples’ number, $\hat{y}_{k}$ represents the predicted target, while $y_{k}$ is the classifier output, and *I* refers to the indicator function. Additionally, we can define precision as

9 $$\begin{aligned} precision = \frac{TP}{TP+FP}. \end{aligned}$$

- **The Cohen Kappa Score (Kappa)**

The Cohen Kappa score evaluates the accuracy that would have resulted from using various estimates. The Kappa score is calculated as^76^.

10 $$\begin{aligned} {Kp=\frac{2 \times (TP\times TN - FN\times FP)}{{(TN+FN)\times (FP+TN)\times (TP+FN)\times (TP+FP)}}}. \end{aligned}$$

- **Matthews Correlation Coefficient (MCC)**

The Matthews Correlation Coefficient (MCC), originally presented by Matthews^77^, is often used for binary classification tasks. The calculation of the MCC is as follows.

11 $$\begin{aligned} MCC ={ \frac{TN\times TP - FN\times FP}{\sqrt{(FN+TN)\times (FP+TN)\times (FN+TP)\times (FP+TP)}}}. \end{aligned}$$

- **F1-Score**

The F1-score is a measure of a model’s accuracy in terms of its precision and recall, as follows:

12 $$\begin{aligned} F1=\frac{TP }{\left( \frac{1}{2} \times FP+\frac{1}{2} \times FN\right) + TP}. \end{aligned}$$

- **Confusion matrix**

For a model with N classes, the confusion matrix is formatted as an $N \times N$matrix, where the rows signify the true class and the columns signify the predicted class, following the standard convention^78^. In addition, the matrix aids in calculating key metrics like the true positive rate $T_{PR}$ and false positive rate $F_{PR}$.

13 $$\begin{aligned} T_{PR}= & \frac{TP}{FN+TP} , \end{aligned}$$

14 $$\begin{aligned} F_{PR}= & \frac{FP}{TN+FP}. \end{aligned}$$

**Fig. 6:**
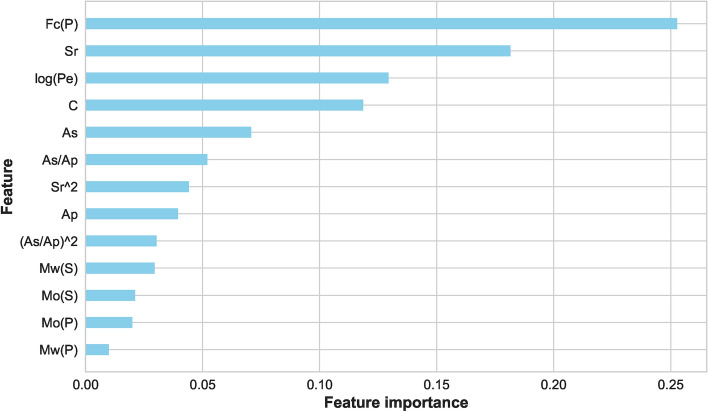
Feature importance plot of all available features.

## Discussion and results

**Fig. 7:**
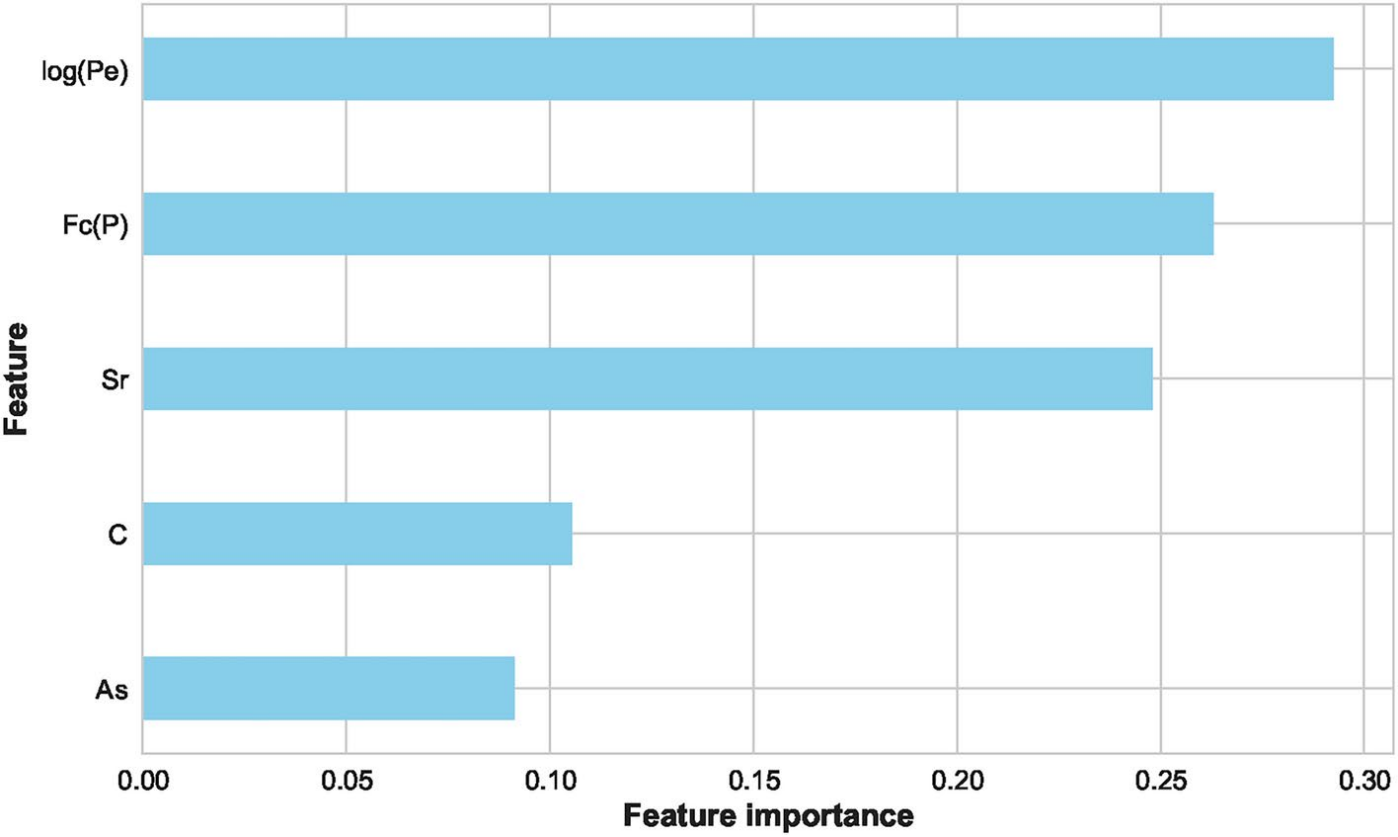
Feature importance plots using the feature selection ‘SelectFromModel’ method.

**Fig. 8:**
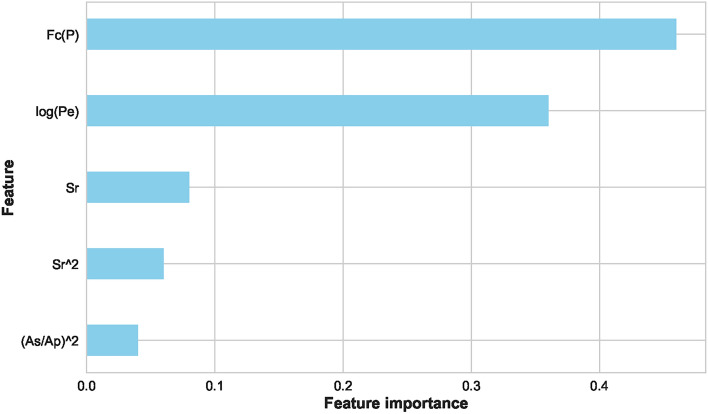
Feature importance plots using the feature selection ‘SelectKBest’ method.

**Fig. 9:**
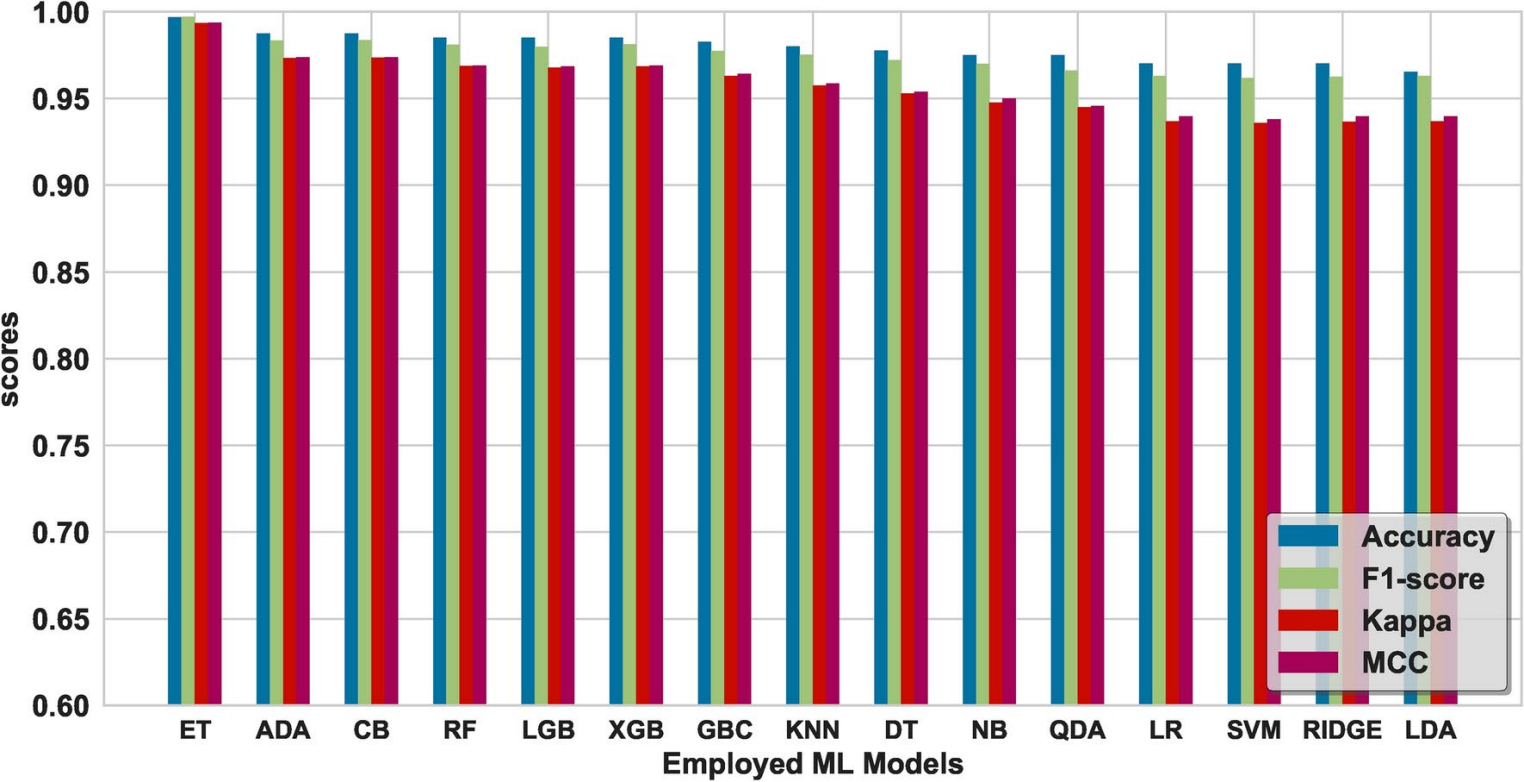
A comparison of Metrics for linear and nonlinear ML models relying on 3 features.

**Fig. 10:**
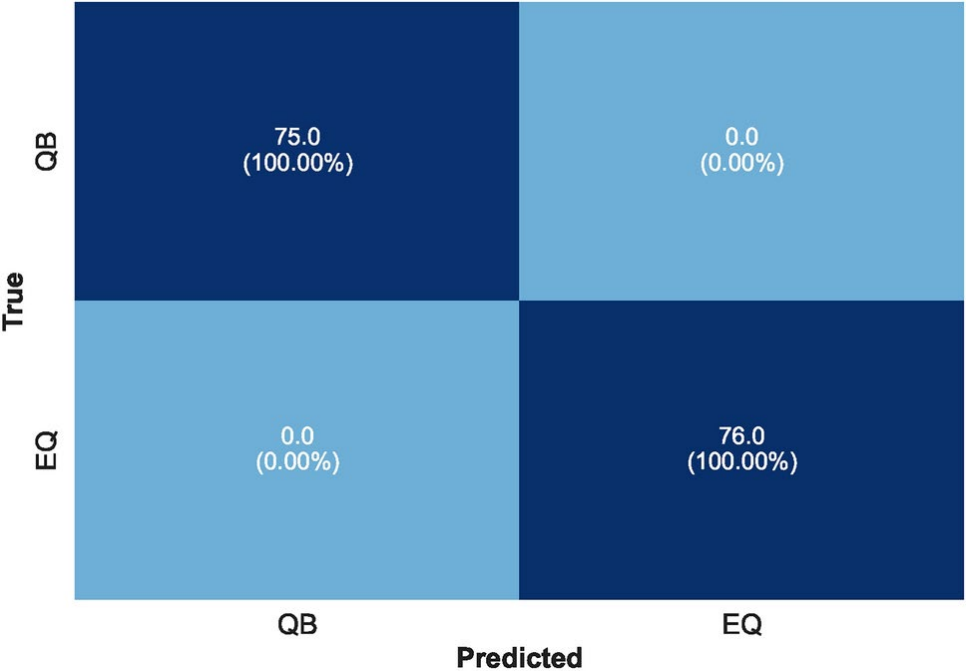
Confusion Matrix of ET classifier.

In this work, we aimed to align with recent developments in seismology research, which has been using machine learning techniques for efficient data processing and signal classification. Many of the previous studies have illustrated the effectiveness of various machine learning models. For instance, some studies worked with linear models like LR and LDA, as well as nonlinear models like NB, RF, and SVM. Several studies depended on the employment of LDA and QDA with spectral parameters of the signals to achieve discrimination^13,15^. Others relied on the use of corner frequency (Fc), seismic moment (Mo), and amplitude ratio (As/Ap) along with LR and NB machine learning models to classify mine events and blasts^22^. While SVM was applied with amplitudes of P-wave and S-wave (As) and (Ap)^25^, it was also experimented with a wavelet filter bank on data from the Aswan area of Egypt^30^. RF model has also been used for the classification of noise and earthquakes and compared to neural networks in some studies^24^. Additional research combined the use of amplitude ratio (As/Ap) and (Sr) with LR, SVM, and NB models^28^. Furthermore, deep learning and convolutional neural networks for discrimination have been deployed recently in Egypt^31,32^. Other studies took another direction by using the catalog parameters along with supervised ML to classify seismic events^18^. Additionally, interesting research has demonstrated the advantage of supervised ML algorithms over unsupervised ones for discrimination studies^27^. While these studies have explored the application of machine learning models to this task using specific models or parameters, we built a comprehensive work that implemented many parameters and various models. This work additionally implied extensive data cleaning steps and the use of two feature selection techniques with the incorporation of feature importance. The available data, which was utilized, covered northern and central Egypt with seven seismic stations. The results of this work are discussed as follows:

As mentioned, several preprocessing steps were performed on the dataset: iterative imputation, data normalization, outlier elimination, and feature engineering. These steps were important to prepare the data for subsequent ML processes. Following preprocessing, we divided the dataset into two parts: ’seen’ data for model training and evaluation and ’unseen’ data reserved for final stage prediction. The ’seen’ data underwent a further split into training and test sets. To evaluate the stability of our models under different conditions, we used two splitting ratios: initially, a 70%−30% split, followed by an 80%−20% split between training and test data. The training set was employed to train various ML models and to tune their hyperparameters. We encountered an overfitting issue with the feature ’Fc(S)’, we had to drop it from the dataset. Using the Scikit-Learn library, various linear and nonlinear ML models were examined using the remaining features. Consequently, the best-performing model at this stage was (LGB) with an accuracy of 99.00%. Then, the feature importance score was plotted for all the available features as depicted in Figure 6. This analysis refers to the degree or extent to which individual features (input variables or attributes) contribute to the performance or predictions of an ML model. In a separate path, two different feature selection methods were applied, ‘SelectFromModel’ and ‘SelectKBest’. With five selected features and an XGB underlying model, this method achieved an accuracy of 99.25% with the (CB) ML model. The feature importance score plot of this method is shown in Figure 7. It was adopted with five selected features and gave the best performance with an accuracy of 99.01% using the (AB) ML model. The feature importance score plot of this method is shown in Figure 8. Through these feature importance analyses, it was clear that some specific ones really matter. These features were identified as the common denominators in the performance of the models and they were Fc(p), Sr, Log(Pe). After dropping other features and applying only these three features, linear and nonlinear ML learning models were adopted. The outperforming model among them was the ET classifier as depicted in Figure 9. It achieved optimal performance through k-fold cross-validation evaluated by average metrics declared in Table 4. An average accuracy of 99.68% resulted through seven folds, making it the optimal model achieved with only 3 features. The confusion matrix is an important tool for assessing a classification problem which indicates how well the predicted classes perform. As shown in Figure 10, the confusion matrix for the optimal model demonstrated accurate classification of ’EQs’ and ’QBs’ as “1” and “0”, respectively.

**Fig. 11:**
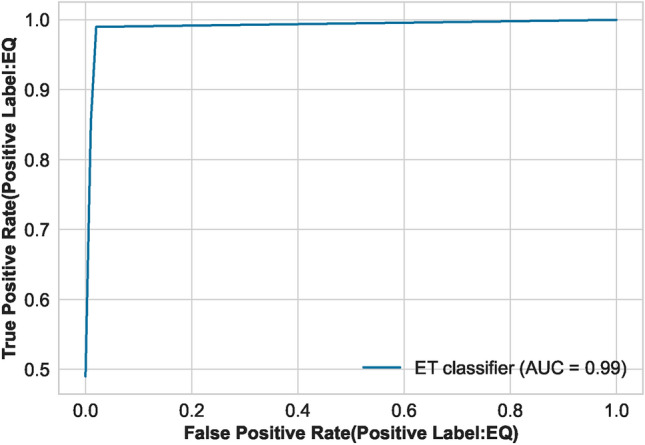
Auc plot of the optimal classifier model.

**Fig. 12:**
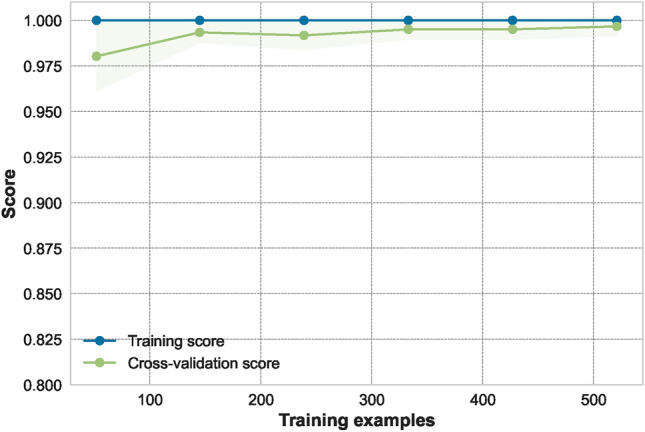
The learning curve of optimal classifier.

**Fig. 13:**
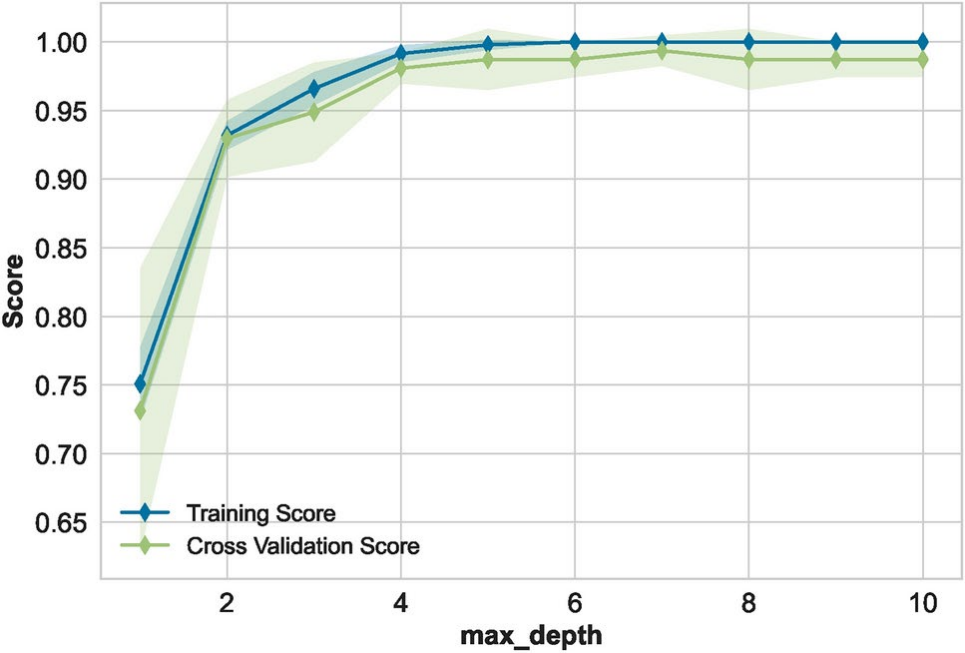
Validation curve of optimal classifier.

**Fig. 14:**
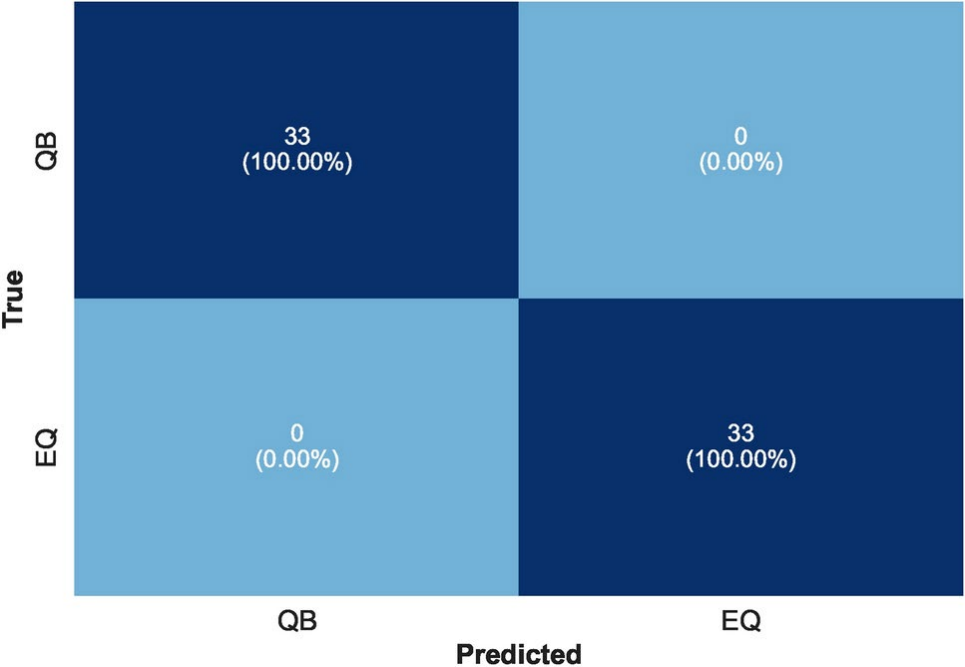
Confusion matrix on unseen data.

The AUC curve of the optimal classifier illustrates its capability to distinguish between the two classes in Fig. 11. The results of the ET classifier, as shown in Fig. 12, indicate that adding more training data is unnecessary, as the training curve has already converged. Furthermore, the validation curve, as shown in Fig. 13, reveals the impact of the “max_depth” hyperparameter during the training process. Finally, we applied the optimally tuned ET classifier to unseen data to evaluate its predictive performance. Its perfect prediction classification is illustrated by the confusion matrix in Fig. 14.

**Fig. 15:**
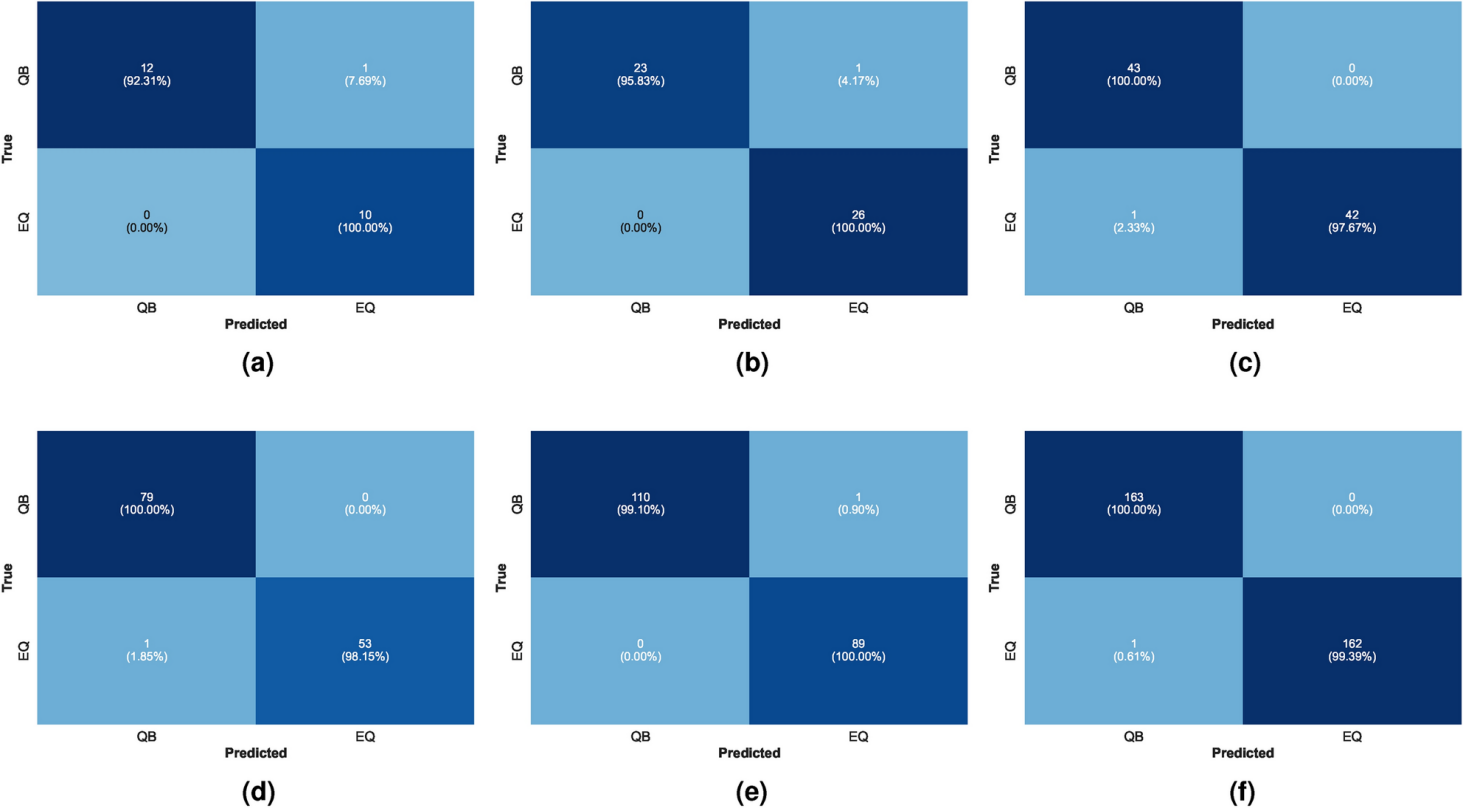
The confusion matrices of ET classifier for 200 samples training set and different population distributions.

Another approach to evaluate the model was undertaken. We used only 200 samples for training and tested the model with different ratios to explore possible population distributions. The 200 samples did not achieve optimal accuracy, as the model generally benefits from larger datasets to stabilize and generalize. There was a misclassification of one event across all trials, which is considered acceptable. The confusion matrices for these trials are illustrated in Fig. 15.

A related study explored a similar approach; it used the input features as pairs and utilized data on a single station level^61^. It achieved the best performance with the XGB model and a pair of parameters (As/Ap) and (Sr), while the pair of (log(Pe)) and (Sr) followed closely. Actually, part of their findings align with ours, in which the parameters (Sr) and (log(Pe)) were shown to be very powerful parameters to be used for discrimination. However, we also experimented with additional parameters that were not used in their study, Fc, Mo, Mw, but were presented in previous studies to be used for classification^12^. In our experiment, the corner frequency appeared to be a powerful parameter for discrimination in this region.

**Table 4:** Metrics of ET classifier.

Metric	Value
Accuracy	99.68%
Recall	100.00%
F1 score	99.70%
Precision	99.67%
MCC	99.37%
Kappa score	99.36%

In this work, the first part of applying all features was to get an overall idea about the behavior of the ML models with all the features and the effect of these features on performance or prediction. It also gave us an idea of how the preprocessing steps and the data cleaning can effectively enhance the ML model’s accuracy. The outlier removal proved to be effective in enhancing the model accuracy, but it may affect the size of the data, so the option of transforming or replacing these values could be a choice to experiment with in the next studies. While the usage of Synthetic Minority Over-sampling Technique (SMOTE) created a balance in the data used, it did not significantly affect the accuracy of the performance. We think with a larger data size and a higher imbalance ratio, it would be more effective, and that is what can be experimented with in future studies. Then, the implementation of the ‘SelectFromModel’ and ‘SelectKBest’ methods, along with Scikit-learn’s feature importance attributes, allowed us to choose the best discrimination parameters from the available data in this region of Egypt. Among the different ML models applied, ET showed the best performance with this kind of data. In order to strengthen this work, we plan to expand the dataset with international data in future research to allow for deeper analysis and a broader understanding of the model’s performance across different regions.

During this study, we faced several limitations and challenges. Identifying and selecting the most relevant features was not an easy process. While feature selection and feature importance techniques improved the model’s interpretability and performance, this may cost computational costs. One of the challenges was the size of the dataset which may limited the ability to thoroughly validate the model’s performance. That is why Cross-validation was used to mitigate this issue to some extent, but in future work, larger-scale validation with independent datasets will be used to confirm the robustness of the findings. Another challenge is the data quality and this was addressed in the preprocessing stage and handling the outliers that could affect the model efficiency. Furthermore, seismic events may evolve over time because of geological changes which require continuous model retraining and updating. Accordingly, more development and adaptation of ML techniques for seismic data are essential to ensure long-term effectiveness and accuracy.

## Conclusion

The discrimination between low magnitude EQs and QBs is one of the main steps to avoid contaminated catalogs which plays a powerful role in the assessment of seismic hazard. The approach that we proposed applied various linear and nonlinear ML models for a binary classification task. We applied feature importance and selection methods across many experiments to identify the most effective features. The best model relying on the most effective features was found with optimal performance. This model was achieved by ET and resulted in 100% classification accuracy on unseen data that was split from the 837 events monitored by seven seismic stations in the region of study. Consequently, this model is recommended to be used in catalogs decontamination and hazard analysis. In our upcoming work, we plan to implement this approach to a wider range of data and different kinds of data like infrasound.

## Data Availability

The datasets used and/or analyzed during the current study are available from the corresponding author upon reasonable request.
